# Time-dependent post mortem changes in the composition of intestinal bacteria using real-time quantitative PCR

**DOI:** 10.1186/1757-4749-5-35

**Published:** 2013-11-25

**Authors:** Sari Tuomisto, Pekka J Karhunen, Tanja Pessi

**Affiliations:** 1 Department of Forensic Medicine, School of Medicine, University of Tampere, Medisiinarinkatu 3, Tampere 33014, Finland; 2 Fimlab Ltd, Pirkanmaa Hospital District, Biokatu 4, Tampere 33520, Finland

**Keywords:** Forensic science, Post mortem microbiology, Fecal sample, Real-time quantitative polymerase chain reaction, Bacterial relative amount, Time-dependent changes

## Abstract

Post mortem or even normal changes during life occurring in major gut bacterial populations are not known. We investigated *Bacteroides* sp., *Bifidobacterium* sp., *Clostridium leptum, Clostridium coccoides*, *Streptococcus* sp., *Lactobacillus* sp. and *Enterobacteriacaea* ratios in 7 fecal samples from healthy volunteers and in 61 autopsies rectum and cecum samples and studied the effect of post mortem time using quantitative real-time PCR. Bacterial ratios in stool samples from volunteers and rectum samples from autopsy cases were similar and did not change significantly up to 5 days post mortem. In cecum, significant post mortem time-dependent differences were observed in ratios of *Bacteroides* sp. (p = 0.014) and *Lactobacillus* sp. (p = 0.024). Our results showed that ratios of *Bacteroides* sp., *Bifidobacterium* sp., *Clostridium leptum*, *Clostridium coccoides*, *Streptococcus* sp., *Lactobacillus* sp. and *Enterobacteriacaea* can be investigated in autopsy rectum samples up to 5 days after death.

## Background

Basic knowledge on the composition of intestinal bacterial populations and changes occurring after death is lacking. Even the normal composition of intestinal microbiota in life is not fully known [1]. Only one study exists in which intestinal bacterial populations have been studied in three elderly women after death using PCR and sequencing [2].

Resident micro-organisms living in the intestinal tract influence host’s normal well-being and physiology including gut metabolism and the regulation of epithelial cell growth [3]. Intestinal microbiota functions as a physical barrier against invading pathogens. It has been suggested that gut microbiota may have a role on the development of diseases, *e.g.* alcoholic liver cirrhosis [4] and atherosclerosis [5]. Detailed bacterial population studies on the intestinal tract have mostly concentrated on fecal samples because they are easy to collect. Intestinal microbiota consists of a large and diverse community containing hundreds of commensal bacterial species [6]. From sequencing libraries of 16S rRNA genes Durban et al. found that two dominant phyla, *Firmicutes* and *Bacteroidetes* accounted for nearly 85% of all sequences in stool samples [7]. Compared to these two major phyla, *Bifidobacterium* genus is present in eight to ten-fold lower numbers [8]. Although *Bacteroides* sp., *Bifidobacterium* sp. and bacteria belonging to the *Clostridium coccoides*–group (cluster XIVa) and *Clostridium leptum*–group (cluster IV) dominate in colon [9,10] there is substantial inter- and intra-individual variation in species composition and distribution [7,11].

This study aimed to investigate ratios of major intestinal bacterial populations in healthy volunteers and in rectum and cecum autopsy samples. Post mortem time-dependent changes were studied in order to see whether autopsy samples can be used for basic research concerning lifetime. Six species: *Bacteroides* sp. (phylum Bacteroidetes*), Clostridium* sp. (Firmicutes*)*, *Streptococcus* sp. (Firmicutes*), Lactobacillus* sp. (Firmicutes), *Bifidobacterium* sp. (Actinobacteria) and *Enterobactericaea* (Proteobacteria) were chosen since they represent the major intestinal bacterial phyla [12].

## Findings

### Study design and results

This study comprises of 61 male cases collected in the Department of Forensic Medicine of the University of Tampere and 7 male volunteers. The selection criteria for the autopsies have been described elsewhere [13]. None of the controls or cases was reported to has been used antibiotics. Deceased had been stored in +4°C within 24 hours after death. Written consent was obtained from the volunteers.

Samples of the autopsy cases were taken from rectum and cecum. All samples were frozen immediately at −80°C until further processing. On the basis of time post mortem the cases were divided into groups: 1–3 days, 4–5 days and >5 days. Demographic characteristics of these groups are shown in the Table 1.

**Table 1:** Demographic characteristics of the study subjects divided by post mortem time

					**Basic cause of death**		
	**N**	**PM mean**	**Age mean (range)**	**BMI mean (range)**	**Heart diseases %**	**Other diseases %**	**Violent deaths (suicide, accident, poisoning) %**
**Autopsy cases:**							
1–3 days	19	2.3	55 (18–79)	29.3 (20.4–42.1)	7 (37%)	4 (21%)	8 (42%)
4–5 days	21	4.5	58 (20–86)	28.4 (18.4–43.6)	10 (48%)	9 (43%)	2 (10%)
>5 days	21	6.5	61 (28–76)	30.7 (21.1–50.3)	15 (71%)	2 (10%)	4 (19%)
p-value			0.373	0.543	0.079	0.086	0.096
Control volunteers	7		45 (26–57)	27.1 (20.8–37.2)			

PM mean = Post mortem mean time.

Fecal samples were weighed to be 150 mg (wet weight). Bacterial DNA was extracted from the samples using Zymo Fecal DNA Kit (Zymo Research Corporation, Irvine, California, USA). The bacterial ratios were determined by RT-qPCR using specific primers and probes (Table 2). The primers and probes for *Enterobacteriacaea* and *Lactobacillus* sp. were designed and confirmed by using BLAST (http://www.ncbi.nlm.nih.gov/) and Ribosomal Database Project (http://rdp.cme.msu.edu/probematch/search.jsp). Specificity and cross reactivity of the designed primers and probes were tested using bacterial cultures from clinical samples [13]. PCR assays were performed with AbiPrism 7000 HT Sequence Detection System (Taqman, AppliedBiosystems, California, USA) with Taqman Environmental MasterMix. Endogen and DNA-free water was used as a negative control.

**Table 2:** Used primers and probes

**Primer and probe**	**Sequence (5′-3′)**	**Reference**
***Bacteroides *** **sp.**		[14]
Forward	TGGTAGTCCACACAGTAAACGATGA	
Reverse	CGTACTCCCCAGGTGGAATACTT	
Probe	GTTTGCCATATACAGTAAGCGGCCAAGCG	
***Bifidobacterium *** **sp** ***.***		[15]
Forward	CGGGTGAGTAATGCGTGACC	
Reverse	TGATAGGACGCGACCCCA	
Probe	CTCCTGGAAACGGGTG	
***Clostridium leptum***		[15]
Forward	CCTTCCGTGCCGSAGTTA	
Reverse	GAATTAAACCACATACTCCACTGCTT	
Probe	CACAATAAGTAATCCACC	
***Clostridium coccoides***		[15]
Forward	GACGCCGCGTGAAGGA	
Reverse	AGCCCCAGCCTTTCACATC	
Probe	CGGTACCTGACTAAGAAG	
***Enterobactericaea***		This study
Forward	GCGGTAGCACAGAGAGCTT	
Reverse	GGCAGTTTCCCAGACATTACTCA	
Probe	CCGCCGCTCGTCACC	
***Lactobacillus *** **sp.**		This study
Forward	GCTAGGTGTTGGAGGGTTTCC	
Reverse	CCAGGCGGAATGCTTAATGC	
Probe	TCAGTGCCGCAGCTAA	
***Streptococcus sp. *** **mainly *Str. mitis-*** group*		[13]
Forward	CCAGCAGCCGCGGTAATA	
Reverse	CCTGCGCTCGCTTTACG	
Probe	ACGCTCGGGACCTACG	
**Universal**		[16]
Forward	TGGAGCATGTGGTTTAATTCGA	
Reverse	TGCGGGACTTAACCCAACA	
Probe	CACGAGCTGACGACA[A/G]CCATGCA	

*This was abbreviated as *Streptococcus* sp. in the text.

The comparative Ct method (ΔΔCt, ΔCt _sample_ – ΔCt _reference sample)_[17], was used where mean values from healthy male volunteers were calculated and used as a reference to determine bacterial relative amount in rectum samples. The differences of the Ct values between the bacteria and the universal bacteria measurement (ΔCt) for each sample were calculated; the comparative Ct (ΔΔCt) for sample and reference samples was calculated. To determine relative amounts of bacteria in cecum samples the rectal sample was used as an inner reference.

Two standard curves were used to determine the total amount of bacteria. Tenfold dilution series of between 33 ng/ml and 0.00033 ng/ml from *E. coli* genomic DNA (ATCC 35401–5) as well as between 10^9^ and 10^5^ colony forming units (CFU) per milliliter from *E .coli* (ATCC 25922) were applied. The amount of CFU or bacterial DNA in the sample was calculated using values from universal measurement and the equation y = slope log (X) + intercept [18].

Statistical analyses were performed with Kruskal-Wallis median test with PASW Statistical Software, version 18 (SPSS Ltd, Quarry Bay, Hong Kong). If P-value was less than 0.05 (considered significant) pairwise Post Hoc comparisons using Mann–Whitney U-test were done.

**Figure 1:**
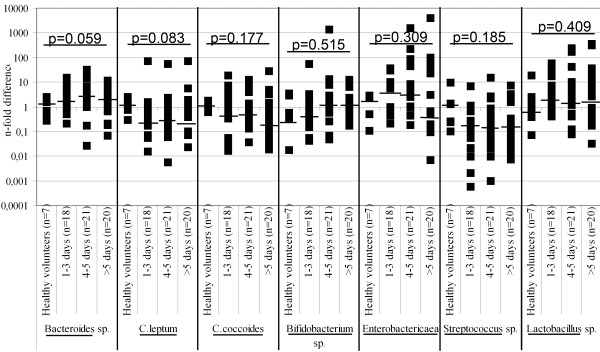
**Relative amounts (n-fold difference) of measured bacteria (*Bacteroides *sp., *C. leptum*, *C. coccoides, Bifidobacterium *sp., *Enterobactericaea*, *Streptococcus *sp. and *Lactobacillus *sp.) in fecal samples of controls and rectum of autopsy cases.** Individual values are presented as boxes, median values with horizontal lines. Comparisons over the groups were calculated using non-parametric Kruskal-Wallis test.

Median values of different bacteria in the stool of healthy controls and in post mortem rectum samples showed no statistically significant changes over post mortem time (Figure 1). In cecum, significant post mortem time-dependent differences were observed over the groups in the relative amounts of *Bacteroides* sp. (p = 0.014) and *Lactobacillus* sp. (p = 0.024, Table 3). There were significantly more *Bacteroides* sp. (p = 0.012) and less *Lactobacillus* sp. (p = 0.015) already in 4–5 days. Statistically significant differences in the total amount of bacterial DNA were seen in healthy volunteers and autopsy rectum samples (p = 0.044, Table 4). In autopsy rectum, the amount of bacterial DNA remained quite stable with time elapsing post mortem except for a high increase observed after day 5 post mortem (p = 0.023). A slightly higher total amount of bacterial DNA (measured as a wet weight) in stool samples donated by the volunteers compared to autopsy rectum samples might be due to lower water concentration in stool compared to rectum without changes in bacterial ratios [19]. Inter-individual variation was great at all time points and in all bacterial measurements.

**Table 3:** The relative amounts (n-fold difference) of measured bacteria in cecum samples compared to rectum samples over post mortem time

		**Bacterial Group**						
		***Bacteroides *** **sp.**	***C. leptum***	***C. coccoides***	***Bifidobacterium *** **sp.**	***Enterobactericaea***	***Streptococcus *** **sp.**	***Lactobacillus *** **sp.**
**All**	**Median**	**0.32**	**0.72**	**1.29**	**1.18**	**0.86**	**2.19**	**0.82**
	**25th–75th**	**0.13–1.06**	**0.41–1.61**	**0.27–3.94**	**0.52–2.66**	**0.09–3.38**	**0.52–7.50**	**0.25–3.61**
1–3 days								
	Median	0.15	0.59	0.64	2.03	1.37	3.56	1.25
	25th–75th	0.01–0.43	0.31–2.94	0.20–4.55	0.84–35.63	0.26–14.77	0.85–35.32	0.46–7.11
4–5 days								
	Median	0.53	1.09	1.27	0.61	0.68	2.28	0.30
	25th–75th	0.17–1.60	0.60–1.81	0.15–2.30	0.26–2.34	0.03–1.85	0.34–8.07	0.16–1.95
>5 days								
	Median	0.53	0.60	2.81	1.03	0.86	1.85	1.09
	25th–75th	0.21–1.45	0.41–1.39	0.59–4.27	0.45–1.61	0.16–7.94	0.27–5.68	0.65–7.84
	p-value	0.014	0.472	0.421	0.054	0.358	0.192	0.024

Results are presented as median and 25^th^-75^th^ interquartile range. Non-parametric median, Kruskal-Wallis-test comparisons between groups.

**Table 4:** The total amount of bacterial DNA in fecal samples

			**N**	ng/g median*	**25**^**th **^**-75**^**th**^	**p-value**^**1)**^	**p-value**^**2)**^
Healthy volunteers	Stool	Control	7	26	9.2-36.7		
Autopsy cases	Rectum	1-3 days	18	8	2.0-53.6		
		4-5 days	21	8	1.7-41.4		
		>5 days	20	42	12.0-124.2	0.044	0.023
Autopsy cases	Cecum	1-3 days	19	51	13-3-94.1		
		4-5 days	21	68	5.1-194.7		
		>5 days	21	48	6.5-113.6		0.982

*1 ng/g corresponds to 4.8x10^10^ colony forming units using *E. coli* as a standard. P-values (over the groups) for ^1)^healthy volunteers and autopsy cases, ^2)^autopsy cases only.

25^th^ -75^th^ interquartile range. Non-parametric median, Kruskal-Wallis-test comparisons over the groups.

## Conclusion

This study showed that relative amounts of major intestinal bacteria in rectum of autopsy cases were similar to stool donated by volunteers and remained quite stable over post mortem time up to 5 days, after which the total amount of bacteria started to increase. In contrast, in cecum significant post mortem time-dependent differences were observed as increase in ratio of strictly anaerobic *Bacteroides* sp. and decrease of facultative *Lactobacillus* sp. due to hypoxia after death. In cecum there is accumulation of undigested nutrients and metabolites produced by bacteria after death, which may be conducive to anaerobic bacterial growth. This study showed that autopsy rectum samples can be used to evaluate major intestinal bacterial populations concerning lifetime up to 5 days after death.

## Competing interests

The authors declare that they have no competing interests.

## Authors’ contributions

ST performed experiments and analyses, helped in collection of the autopsy samples and wrote the manuscript. PK was the iniator of the project and group leader and participated in writing the script. TP was the guarantor of the microbiological part of the study, designed the sample collection and experiments, and participated in writing the manuscript. All authors read and approved the final manuscript.
